# Heterogeneity of Lung Phagocytes and Clearance of Apoptotic Cells in Lung Injury and Repair

**DOI:** 10.1055/a-2675-2564

**Published:** 2025-09-04

**Authors:** Stephanie M. Bersie, Alexandra L. McCubbrey

**Affiliations:** 1 Toxicology Graduate Program, University of Colorado Denver Skaggs School of Pharmacy, Aurora, Colorado; 2 Department of Medicine, National Jewish Health, Denver, Colorado; 3 Department of Medicine, University of Colorado Anschutz Medical Campus, Aurora, Colorado

**Keywords:** efferocytosis, apoptotic cell clearance, lung, macrophage, nonprofessional phagocyte, repair

## Abstract

Poor repair following lung injury is a significant cause of morbidity and mortality. Clearance of apoptotic cells, termed efferocytosis, has emerged as a key process that can influence repair outcomes and facilitate successful repair. Although prior literature has focused on efferocytosis by macrophages, evidence is emerging that nonprofessional phagocytes, including fibroblasts and epithelial cells, may play critical roles in efferocytosis during tissue repair. This review summarizes existing knowledge of different lung phagocytes that can participate in efferocytosis, evidence linking efferocytosis to lung health and tissue repair, and discusses factors that may inhibit or redirect efferocytosis to promote mis-repair. A deeper understanding of how the integrated landscape of lung phagocytes participates in efferocytosis will likely provide significant insight into repair and mis-repair processes.

Lung injury is broadly associated with cell death, both of damaged structural cells and of short-lived recruited immune cells. Early work established this as primarily apoptotic cell (AC) death. 1 2 3 4 5 Excellent reviews have been published that discuss apoptosis and how it compares to other modes of cell death. 6 7 Briefly, apoptosis is a specific type of caspase-mediated death. It can be initiated at the cell surface or mitochondria, but both routes converge to activate caspase-3. Caspase-3 cleaves proteins throughout the cell and triggers surface exposure of phosphatidylserine (PS), a lipid that is normally sequestered on the inner leaflet of the plasma membrane. PS is recognized by surface receptors on phagocytes, enabling AC to be cleared prior to membrane rupture by efferocytosis. This key feature of PS-exposure prior to loss of membrane integrity allows AC clearance before the release of inflammatory intracellular contents. This has led to apoptosis being termed “immunologically silent.” In actuality, apoptosis has profound immune impacts that are predominantly anti-inflammatory and pro-repair. 5 AC release proteins and metabolic mediators during apoptosis with paracrine anti-inflammatory effects, and actual efferocytosis triggers pro-resolution and pro-repair programming in engulfing phagocytes. 8 9 10 11 This has been connected with successful lung repair. However, it is now recognized that the type of phagocyte, the type of AC, and broader environmental context all influence how efferocytosis links to resolution outcomes. In certain contexts, efferocytosis may, in fact, participate in driving dysfunctional repair. To harness efferocytosis therapeutically, it will be critical to understand these contexts.

Basic mechanics of efferocytosis: The mechanics of efferocytosis are well-understood. 12 13 Briefly, AC release chemoattractants that direct the movement and migration of nearby phagocytes. In some tissues, phagocyte cell bodies have been shown to remain stationary while processes extend to interact with AC. 14 AC binding involves integrin-driven tethering and adhesion that enables the interface of recognition receptors with PS or bridge molecules coating the AC surface. Key receptors that will be mentioned in this review include Axl, MerTK, and Tyro3, which recognize PS through bridge molecules Gas6 and ProS. It is believed that successful tethering and recognition generate a phagocytic synapse with specific zones, enabling Rac1-dependent actin cytoskeletal rearrangement to form a phagocytic cup for engulfment. 15 Once internalized, this AC phagosome is acidified through intracellular machinery shared with LC3-associated phagocytosis, maturing into the phagolysosome. This degrades the AC, releasing metabolites that are used to fuel cellular processes and new protein synthesis. Efferocytosis has been shown to profoundly impact macrophage metabolism, programming, and function, with changes in metabolism strongly linked to pro-resolution immune function. 10 11

Negative regulation of efferocytosis: Tethering and recognition of AC can be antagonized by “don't eat me” signals expressed by living cells in the body. 16 17 The most well-described negative regulator of efferocytosis is CD47 on target cells, which is recognized by SIRP-α on phagocytes, activating SHP phosphatases and disrupting the formation of the organized phagocytic synapse and phagocytic cup. However, several recent advances have been made expanding our understanding of an underappreciated “don't eat me” signal, the glycocalyx.

The membranes of most cells are coated by a mesh of glycans, glycosylated lipids, and glycosylated proteins, together termed the glycocalyx. 18 It has been recognized for decades that glycans and glycoproteins in the cell membrane are lost or modified on cells that are undergoing apoptosis. 19 20 21 22 This has been connected to enzyme-driven shedding and exposure of normally intracellular glycan moieties and has been shown to promote increased engulfment of AC. Recent work has revisited these findings and provided new insight. Work by Imbert et al demonstrated how a combination of both target and phagocyte glycocalyx forms a bidirectional barrier to engulfment extending past phagocytic receptors to prevent cell–cell recognition. 23 This was not specific to but included recognition of AC. Intriguingly, they found that macrophages innately synthesized this barrier or changed its thickness with an environmentally acquired coating. Additional works by Le et al and Drexhage et al identified novel mechanisms by which ACs manipulate the glycocalyx to enable phagocytic recognition. 24 25 In one paper, ADAM10 was identified as a sheddase that is activated by caspase-3 and PS exposure on AC to remove specific glycocalyx mucins from apoptotic T cells. 24 In the second paper, cytoskeletal rearrangement involved in bleb formation was shown to displace the glycocalyx from AC bleb regions independent of shedding, redistributing CD44 and hyaluronan away from blebs to facilitate cell–cell contact between early AC and engulfing phagocytes. 25 These findings are intriguing to consider, given the rise in endothelial and epithelial glycocalyx shedding following lung injury. 26 It is possible that injury-triggered cleavage of the glycocalyx alters phagocyte-AC interactions.

## Subsets of Lung Phagocytes and Current Literature Describing Their Efferocytosis

The majority of efferocytosis in the body is performed by macrophages, but AC clearance by nonmacrophages can be critical. Processes including mammary involution and daily photoreceptor outer rod clearance rely on AC engulfment by “amateur” or “nonprofessional” epithelial phagocytes. In fact, most structural cells have the capacity for recognition and engulfment of dead cell material, albeit often to a more limited degree than “professional” phagocytes such as macrophages. 27 An unanswered question remains: what do structural cells do after they engulf AC? Nearly a decade ago, Bosurgi et al put forth that AC can be conceptualized as a “context cue” that fundamentally informs macrophages about the need for repair and unlocks pro-resolving programming. 28 It is intriguing to hypothesize that the answer for structural cells is the same and that sensing cell death acts to license specific repair programming in structural cells. With new techniques for lineage tracing and tracking efferocytosis in vivo, the field is poised to consider how the landscape of pulmonary phagocytes can jointly participate in sensing and clearing AC and determine how efferocytosis impacts each phagocyte subset. Given their high engulfment avidity, professional subsets likely outcompete nonprofessional subsets for AC signals in healthy settings, but this may be unbalanced in disease. Below, existing data on five types of lung phagocytes will be discussed to explain what is known about each subset and efferocytosis. This review does not address lung granulocytes, but they are likely to be another family of phagocytes that should be considered in this context. 29 30

Macrophage subsets: Healthy lungs contain resident macrophage populations in the airspace lumen (alveolar macrophages, AM) and tissue (interstitial macrophages, IM). Lineage tracing in mice has shown that resident AM and IM arise during embryogenesis and will self-renew throughout life unless perturbed. Sequencing studies have further identified two IM subsets, best marked in mice and humans by expression of FORLB. FOLRB + IM, sometimes called perivascular IM or TLF macrophages, are particularly enriched around vessels, while FOLRB − IM are enriched around the airways and between alveoli. 31 32 33 34 35 36 Single-cell sequencing has identified heterogeneity within healthy AM populations, most notably by the presence of rare monocyte-like AM and interstitial-like AM in humans. 37 It is believed that the majority of AM reside within the alveoli, where they receive identity-defining granulocyte-macrophage colony-stimulating factor (GM-CSF) from alveolar epithelial type II cells (ATII) and participate in the recycling of lung surfactant. Whether specific AM subpopulations can stably reside outside of the alveoli in larger airways or bronchoalveolar duct junctions remains an open question.

Upon perturbation, as occurs in lung injury, monocytes are recruited to the lungs. In the airspace, they differentiate into recruited AM; in the tissue, they differentiate into recruited IM. During early inflammation, recruited AM are distinct from resident AM, expressing unique transcriptional programs and clear surface markers reflective of their monocyte origin. 38 Over time, recruited AMs adopt a resident AM-like profile until they are near-indistinguishable. 39 Within recruited AM, further subsets have been identified, with GPNMB +/SPP1+ subsets particularly connected to tissue repair and fibrotic mis-repair. Studies of recruited IM are scant; murine studies examining LPS and bleomycin-induced lung injury found that they rapidly overlapped with FOLRB − IM in both surface markers and transcriptional programming. 31 40 Lung macrophage development and subsets have been the subject of many recent reviews. 41 42 43

AM efferocytosis: Multiple reviews have been published on AM efferocytosis. This review focuses on what has been recently published about murine AM subsets. Very little data exists for human AM subsets; the only point of note is that human interstitial-like AM express extremely high MerTK. 37 Most, but not all, murine studies report that resident AM have a greater capacity for efferocytosis than recruited AM. 44 45 46 47 Differing results likely reflect variation between recruited AM maturation in studies, as expression of phagocytic receptors, including MerTK and Axl, is gained with maturity. 44 This is supported by a study by Gibbings et al contrasting resident and recruited AM efferocytosis 1 month following recruitment; no difference in efferocytosis was observed between AM subsets at this time. 39

Two recent studies were designed to isolate the impacts of resident AM efferocytosis on resolution after lung injury. A publication by Guttenberg et al demonstrated that depletion of resident AM with clodronate prior to ozone injury led to higher numbers of apoptotic neutrophils persisting in bronchoalveolar lavage fluid (BALF). 48 In addition, excessive apoptotic neutrophils were observed in lavage from MerTK-knockout mice after injury at a time point preceding monocyte recruitment. This coincided with elevated pro-inflammatory BALF cytokines, including IL-6 and MCP-1. Another publication by Chakraborty et al examined the expression of efferocytosis receptors on AM following repeat LPS exposures. 49 They noted enhanced expression of MerTK on SiglecF-high AM in response to a second LPS exposure, putatively resident AM, although lineage tracing was not used to confirm. They found that this trained MerTK induction was dependent on Klf4, improved clearance of apoptotic neutrophils from lavage, and accelerated tissue resolution. Both studies point toward resident AM as key mediators of efferocytosis that clear AC and regulate early inflammation after lung injury. Current literature describing recruited AM suggests that they may become increasingly important for AC control as they mature. In a study by Liang et al, overexpression of CCL2, which drives monocyte recruitment to increase recruited AM, led to decreased numbers of apoptotic neutrophils after lung injury, with a minor difference observed by 3 days and significant acceleration in clearance evident 7 days after injury. 46 In a more recent study by Nepal et al, the concept of mature versus immature recruited AM was modeled with bone marrow-derived macrophages (BMDMs). Intratracheal instillation of live BMDM that were matured ex vivo to express high Gas6 could accelerate neutrophil clearance in the lungs, while control BMDM could not. 50

IM efferocytosis: Far less is known about IM than AM. Early work in mice confirmed that IM are phagocytic and engulf bacteria along with carboxylated latex beads, which are often used as a surrogate for AC. 51 52 Sequencing data show that both IM subsets highly express MerTK, Axl, and other AC recognition receptors. 31 51 FOLRB + IM show increased expression of additional phagocytic recognition receptors, CD36 and Tim4, while FOLRB − IM show increased expression of MHCII molecules. 31 This may imply functional differences in the ability of each subset to cross-present cell-associated antigens, but this remains unknown. Intriguingly, FOLRB + IM showed higher engulfment of intra-nasally administered material than FOLRB − IM, even though ex vivo engulfment rates were similar, suggesting unique access of FOLRB + IM to material in the airspace. 51 There is some suggestion from a recent report examining efferocytosis following influenza that IM may have a particular role in engulfment of apoptotic ATII cells in vivo. 53 However, this contrasts with a report of robust ATII efferocytosis by AM in a KO mouse with emphysema. 54 It may be that the fate of apoptotic ATII cells depends on the type of lung injury. Notably, lung macrophage subsets have distinct transcriptional licensing for many repair factors. 31 It is intriguing to consider that efferocytosis by one macrophage type over another may be needed to tailor repair outcomes to the type of injury.

Suppression of macrophage efferocytosis in the alveolar space: It is notable that even with robust expression of PS-recognition receptors, fewer than half of AM show capacity for efferocytosis. This is consistently below the capacity reported for peritoneal macrophages under equivalent conditions. 47 55 Multiple studies have shown that factors in the alveolar environment, including GM-CSF and surfactant proteins, actively inhibit efferocytosis. 47 56 This has led to speculation that suppressing efferocytosis is part of a delicate balance, weighing the prioritization of AC removal against other key AM functions, including pathogen control and surfactant recycling. For example, multiple studies have reported that efferocytosis antagonizes the clearance of S. pneumoniae in the lungs and is a mechanism of spread for other bacteria and viruses. 57 58 59 60 61 However, apoptosis and efferocytosis are also critical aspects of pathogen control that can deprive pathogens of their niche, facilitate the generation of an adaptive immune response, and limit immunopathology. 62 63 64 Thus, the concept of advantageous efferocytic restriction should not be considered in conflict with robust data showing that AM efferocytosis is critical for lung repair, as will be discussed in detail below. However, a therapeutic goal to simply “improve” efferocytosis may unbalance other key phagocyte functions.

Dendritic cell (DC) subsets: Nomenclature for DC remains inconsistent, but there are three main subsets found in healthy lungs: DC type 1 (cDC1, also called CD103 + , BATF3 + , or BDCA3 + ), DC type 2 (cDC2, also called CD11b + , IRF4 + , or BDCA1 + ), and plasmacytoid DC (pDC). Current evidence supports that they arise from a shared CX3CR1+ bone marrow progenitor, not monocytes. 65 However, monocyte-derived “inflammatory” DC can be recruited during injury, marked by the expression of Ly6C in mice or CD14 in humans. Detailed information on lung DC subsets can be found in recent reviews. 66 67

DC efferocytosis: Although efferocytosis by macrophages has been the dominant focus of research, early studies established that efferocytosis was also performed by cDC1 (marked by CD8-α expression), with minimal efferocytosis by cDC2 or pDC. 68 69 70 A decade later, similar data were observed by Desch et al in the lungs: cDC1 DC (also known as CD103+ or Batf3 + ) had a heightened capacity for efferocytosis, specifically migrated to the draining lymph nodes, and facilitated cross-presentation of cell-associated antigens to activate CD8+ T cells. 71 However, with advancements in the understanding of DC development and studies of DC in various states of activation, researchers have begun to realize this delineation may be more complex. All DCs express many phagocytic recognition receptors, including integrins, CD36, and PS-receptors. 72 73 74 75 Very few, such as Tim4, are specific to or highly enriched on cDC1. A recent kidney study by Ruben et al found that pDC capacity for efferocytosis could be induced by infected AC requiring GM-CSF and TLR9. 76 This is part of a growing body of literature showing that infected versus noninfected AC have differential impacts on DC, a biology that is likely to be extremely important for pathogen responses in the lungs. 77

Most literature supports that, similar to macrophages, the default DC response to efferocytosis is immune suppression and tolerance. However, it is clear that infected AC or genetic loss of DC regulatory pathways can initiate maladaptive cross-presentation with T cell activation. 78 79 This suggests that particular cues during injury could quickly convert efferocytosing DC to antagonists of resolution. When considering the role of DC within the pulmonary system, it is important to note that DC have low numbers and low efferocytosis efficiency when compared with lung macrophages. However, it is clear that DC can engulf AC and that their function is dramatically altered by efferocytosis, placing them as undeniable but understudied participants in the lung response to AC.

Epithelial cell subsets: Single-cell RNA-sequencing has significantly advanced our understanding of epithelial cell subsets in the lungs, with at least nine subsets of bronchial epithelial cell identified in the airway (club cells, ciliated cells, tuft cells, goblet cells, deuterosomal cells, pulmonary neuroendocrine cells, basal cells, suprabasal cells, and ionocytes) along with alveolar type I (ATI) and alveolar type II (ATII) cells in the alveoli. Following injury, intermediate state cells are regularly observed as part of ATII to ATI differentiation and epithelial repair. 80 Detailed discussion of epithelial cell subsets can be found in this recent review. 81

Epithelial cell efferocytosis: There is evidence that some type of bronchial epithelial cell can perform efferocytosis. Early studies found that human bronchial epithelial cells engulfed AC using integrin and PS-dependent recognition. 82 83 However, a key caveat to this work is that cells were grown in submerged cultures, and thus did not focus on a specific subset or replicate normal epithelial physiology. More recent studies of murine lungs by Shibata et al show low-level MerTK expression in healthy airway epithelial cells, which could mediate PS-dependent AC recognition. 84 Intriguingly, they also note MerTK (and to a lesser extent Axl) expression in the alveoli following infection, spatially consistent with expression on both ATI and ATII cells. Whether alveolar epithelial cells can perform efferocytosis remains unknown; however, these data suggest that they may at least gain the ability to sense AC following tissue injury. Of note, while many immortalized lung epithelial cell lines are capable of performing efferocytosis, it is unlikely to be an accurate representation of normal epithelial cells, since high expression of MerTK and Axl or gain-of-function mutations are well-established in epithelial tumor cell lines. 85

One published study has assessed the physiologic importance of lung epithelial cell efferocytosis. It also comprises the single report measuring efferocytosis by intact bronchial epithelial cells. Juncadella et al used a doxycycline-inducible CCSP-Cre mouse strain to delete Rac1 in bronchial epithelial cells. 86 Upon intra-tracheal instillation of AC to the lungs, they reported that IL-10 and TGFβ expression in BALF, along with bronchial epithelial cell efferocytosis of instilled AC, were all decreased in Rac1-deficient animals. Epithelial cells were simply defined as CCSP-expressing by a reporter, so insight into epithelial cell subsets was not gained. Collectively, although data are scant, this supports that bronchial epithelial cells have the capacity for efferocytosis and a largely unexplored role in setting lung immune tone in response to AC, but many questions remain.

Endothelial cell subsets: Single-cell RNA-sequencing has determined that the lungs contain unique lymphatic, artery, vein, and capillary endothelial cell types, with two subsets each of vein and capillary cells for a total of six endothelial subsets. 87 Capillary subsets are the general capillary endothelial cell and the aerocyte, which is a very thin cell specialized for gas exchange in partnership with ATI epithelial cells. 88

Endothelial cell efferocytosis: Though efferocytosis has not been directly assessed, there is evidence that at least some lung endothelial cell subsets are capable of sensing and being reprogrammed by AC. Li et al demonstrated that human lung microvascular endothelial cells express Axl and MerTK (with minor expression of Tyro3). 89 In this study, loss of MerTK increased endothelial barrier permeability and neutrophil transepithelial migration in vitro, but efferocytosis was not examined. Studies of nonlung lymphatic, aortic, and umbilical vein endothelial cells have reported efferocytosis of various target cells, including aged red blood cells in vitro. 90 91 92 93 94 95 These study designs did not address how efficiently endothelial cells perform efferocytosis, but did show upregulation of MerTK, secretion of IL-8, and changes to endothelial migration after co-cultures with AC. While future studies are needed, this data suggests that lung endothelial cell function could be altered by signaling in the presence of uncleared AC. Given their key barrier function and thin shape, it may be unlikely that they participate significantly in AC clearance.

Fibroblast subsets: At least three types of fibroblasts have been identified by transcriptional programs and localize to specific regions of the lungs: alveolar, adventitial, and peribronchial. 96 In addition, Cthrc1+ fibroblasts have been described after injury and are associated with fibrotic tissue, which is believed to arise from alveolar fibroblasts. 97 98 99

Fibroblast efferocytosis: No studies have directly assessed efferocytosis by lung fibroblasts, but fibroblasts are known to express multiple PS-receptors, bridge molecules, and integrins involved in efferocytosis. Intriguingly, fibroblasts from idiopathic pulmonary fibrosis (IPF) lungs have high Axl, coinciding with loss of low-level MerTK. 99 Axl/Gas6 signaling in fibroblasts has been linked to fibroblast proliferation and the development of fibrosis after bleomycin-induced lung injury. 99 100 Importantly, Axl has been shown to require PS along with Gas6 for kinase activation, 101 suggesting that AC or other dead cells generated during tissue damage may be required for this Axl-fibroblast-fibrosis connection. Fibroblast efferocytosis has been examined in other tissues and has been shown to occur in the heart and skin, where it is linked to successful repair. 102 103 Collectively, these data show that lung fibroblasts express the machinery necessary to interact with AC and suggest that they are functionally altered by AC in their environment. Given that in the lung, this appears to be connected with fibrosis, a key benefit of efficient macrophage efferocytosis following injury may be in removing AC to moderate the activation of fibroblasts.

## Links Between Efferocytosis and Lung Disease, Lung Repair, and Lung Fibrosis

Understanding the importance of efferocytosis has been heavily informed by the observation that defective AC clearance is observed during autoimmunity, atherosclerosis, failed wound healing, liver injury, transplant rejection, and neurodegeneration. 11 104 105 106 107 Indeed, impaired AM efferocytosis has been observed in many lung diseases, perhaps triggered by mucus obstruction and prolonged airway damage. 108 109 As discussed below, defective efferocytosis is associated with oxidative stress, mitochondrial dysfunction, destruction of lung structural integrity, aberrant repair after acute lung injury (ALI), and failure to resolve inflammation. 110 111 However, there is also a suggestion that poor efferocytosis in IPF may reduce fibrotic processes. Of note, a major caveat to existing studies is their exclusive focus on AM; contributions of other lung phagocytes remain unknown.

Chronic obstructive pulmonary disease (COPD): Elevated numbers of apoptotic airway epithelial cells, infiltrating T cells, monocyte-derived macrophages, and neutrophils are observed in the lungs of COPD patients, consistent with defective clearance. 112 113 114 115 Disease severity has been correlated with the degree of AM efferocytic impairment compared with healthy controls, 116 and in a mouse model of emphysema, inhibiting efferocytosis worsened lung destruction. 117 Poor efferocytosis has been proposed as a major disease-driving mechanism in COPD.

Asthma: Phagocytes isolated from patients with both allergic asthma and neutrophilic asthma have significantly reduced efferocytic capacity compared with healthy controls. 118 119 120 There are hints to potential mechanisms: downregulation of Axl was observed in AM from patients with asthma, while a murine model of allergic lung inflammation showed decreased Rac1 expression. 121 122 One study found efferocytic failure in neutrophilic asthma to be more pronounced. 123 Low efferocytosis has been linked to persistent inflammation: MerTK-knockout mice showed delayed resolution in a murine model of allergic inflammation, and human AM from asthma patients had decreased induction of anti-inflammatory PGE-2 and 15-HETE in response to AC. 118 119 The link is less well-established than COPD, but poor efferocytosis is believed to contribute to asthma.

Cystic fibrosis (CF): The CFTR mutation has been connected to elevated RhoA expression, a negative regulator of efferocytosis. 124 Elevated neutrophil elastase (NE), released by infiltrating neutrophils, is also a hallmark characteristic of CF, and studies have identified NE-mediated cleavage of PS receptors. 125 Further, CF mouse models have shown HMGB1, which can inhibit efferocytosis via PS binding, to be elevated in BALF. 126 These data implicate multiple modes of impaired AC clearance in CF patients, which is presumed to amplify their lung inflammation.

ALI and acute respiratory distress syndrome (ARDS): The preponderance of data supporting that efferocytosis regulates repair in the lungs comes from studies of ALI and ARDS. A recent publication by Mahida et al found that ARDS-sepsis patients displayed markedly impaired AM efferocytosis, and that low efferocytosis was correlated with decreased survival. 127 Proposed mechanisms for suppression of efferocytosis in patients with ARDS include elevated HMGB1 in the alveolar space, high RAB11A in recruited monocytes, impaired activity of AM HSD-1, and depressed expression of CD36 and MERTK. 128 129 130 131 Numerous murine studies using diverse models of lung injury provide further conceptual support that AMs clear AC from the airspace, and suppression of AM efferocytosis leads to prolonged inflammation and poor health outcomes. 48 49 50 132 133 134 135 136 Exactly how efferocytosis promotes resolution and repair in the lungs is likely multifaceted. There is evidence that the beneficial effects of efferocytosis include activation of AM PPARγ and increased secretion of HGF and TGFβ. From an interventional standpoint, multiple studies have shown improved AM efferocytosis following intratracheal instillation of recombinant Gas6, accompanied by reductions in proinflammatory cytokine production. 137 138 In addition, instillation of apoptotic T cells has been shown to accelerate resolution after ALI, which intriguingly suggests that in some injury settings AM may lack target AC to “feed” efferocytosis. 132 This concept of lacking AC has some support in human patients with ARDS; a recent study found that ARDS neutrophils commit to NET formation rather than apoptosis. 128

Since ARDS is commonly a consequence of severe infection, it is important to emphasize that infected AC have been reported to induce inflammatory cytokine production rather than promote resolution when engulfed, although this has not been specifically confirmed for lung phagocytes. 131 139 140 This identifies two reasons why correcting defective AM engulfment in ARDS may not be the only roadblock to harnessing efferocytosis-driven resolution: (1) immune cells in the alveoli may not be undergoing normal apoptosis, and (2) engulfment of any infected AC would likely skew phagocyte reprogramming away from resolution and back to inflammation. Correcting efferocytosis remains an attractive therapeutic target to improve outcomes in ARDS, but these studies illuminate the complexity of that goal.

IPF: Unlike the other diseases discussed, the sum of data assessing efferocytosis in fibrotic disease suggests that depressed efferocytosis may be protective in IPF. Aberrant cell death is a clear hallmark of IPF. While alveolar epithelial apoptosis is observed in many lung diseases, it is particularly high in IPF and accompanied by apoptotic resistance in fibroblasts, a biology that has been directly connected to disease pathology. 141 142 143 144 Decreased efferocytosis has also been reported in IPF, 145 which at the time was presumed to contribute to disease pathogenesis. However, since that publication, high MerTK and Axl expression have been linked to worse fibrosis in humans and mouse models, suggesting that better efferocytosis may, in fact, contribute to pathogenesis. 146 147 This may critically relate to structural cell recognition of dead cells via Axl and MerTK. However, it may also relate to the specific presence of apoptotic epithelial cells. In a recent publication, Kim et al found that intratracheal instillation of apoptotic epithelial cells, but not apoptotic T cells, led to high levels of TGFβ in BALF and the development of lung fibrosis. 148 This is notable because two other studies reported that instillation of apoptotic T cells given after bleomycin-induced lung injury protected mice from the development of fibrosis. 149 150 This implies that pro-fibrotic reprogramming occurs specifically in response to engulfment of apoptotic epithelial cells, which also has conceptual support from a recent paper examining efferocytosis of immune versus structural AC. 151 Along with ARDS, IPF highlights our evolving understanding regarding the complexity of how efferocytosis intersects with repair in the lungs and echoes a clear theme in our understanding of fibrotic lung disease: the processes underpinning fibrosis are closely related to those necessary for repair.

## Environmental Factors Influencing Efferocytosis

When considering the functionality of efferocytosis in the human lung, it is important to recognize that environmental exposures can dramatically impact phagocyte functions. Systemic factors reported to alter macrophage efferocytosis include high salt diet, alcohol use or abuse, organophosphate exposure, broad-spectrum antibiotics, and common respiratory pharmaceuticals such as glucocorticoids. In addition, lung phagocytes will be uniquely susceptible to the impacts of inhaled materials. As discussed further below, many inhaled exposures suppress efferocytosis, which may contribute to persistent inflammation and promote mis-repair following lung injury. 152 Equally intriguing but unexplored is to consider how AC, laden with various environmental exposures, may impact phagocyte reprogramming. As with infected AC, particulate in AC may provide a co-cue that subverts normal pro-resolution programming when engulfed.

Cigarette smoke (CS): This is a leading cause of COPD but has been independently associated with impaired AM efferocytic capacity. 153 154 155 156 157 158 159 Several studies have found that AM from patients with or without COPD who are active smokers have worse efferocytosis than nonsmokers or COPD patients who are former smokers. 154 156 160 Murine studies have shown that longer CS exposure leads to more persistent suppression of AM efferocytosis following withdrawal. 161 162 There is significant evidence that the trigger is CS-driven ER and oxidative stress; efferocytosis was preserved in smoke-exposed mice that were genetically resistant to oxidative stress or treated with GM-CSF, macrolides, or the antioxidant procysteine. 156 157 161 163 Wood smoke has also been reported to inhibit efferocytosis, although mechanisms are unknown. 164

Vaping and E-cigarettes: These were promoted as a safer alternative to smoking, but the health hazards of vaping are becoming better understood. Several studies have identified impaired AM efferocytosis and bacterial phagocytosis following E-cigarette exposure. 165 166 167 The severity of impact on efferocytosis and other cell processes is dependent upon the chemical profiles of solvents and flavor additives in vapor. 165

Heavy metals: Cadmium, arsenic, and beryllium are known to contribute to respiratory dysfunction and are common components of mixed particulates, including CS, E-cigarette vapor, and industrial exhaust. 152 Though studies are limited, metals have been linked to dysfunctional efferocytosis. One study examining exposure to WC-Co dust, a common metal alloy composed of tungsten carbide/cobalt, identified increased apoptosis concurrent with impaired macrophage efferocytosis. 168 Another study demonstrated that beryllium bound to PS on AC and prevented recognition by many PS receptors, blocking uptake of aged erythrocytes. 169

Crystalline silica: This is commonly used in industrial manufacturing and can trigger severe fibrotic lung disease called silicosis. Inhalation is also a trigger for autoimmune diseases such as systemic sclerosis and lupus, which have been linked to silica-driven impairment of efferocytosis. 170 171 172 Silica induces RhoA activation and perturbs MerTK and Axl expression. As strong evidence that efferocytosis is acting to mitigate damage in silicosis, a recent murine study showed that loss of Axl or MerTK led to persistent inflammation, and Axl-knockout led to a 40% decrease in survival rate. 173

## Conclusion

Harnessing the pro-repair and pro-resolution properties of efferocytosis is an attractive therapeutic angle to augment lung repair, but there is emerging evidence that context matters for these positive effects. Factors that inhibit efferocytosis or redirect phagocyte programming by efferocytosis may both lead to mis-repair. Many different types of cells will die during tissue injury, and many different types of phagocytes may be involved in their clearance. To understand how efferocytosis impacts lung repair, it is essential to identify (1) how the integrated landscape of lung phagocytes performs efferocytosis, particularly nonprofessional phagocytes, (2) whether the identity of AC such as structural versus immune alters repair responses, and (3) how inhaled exposures to pathogens or particulates change the efficiency and perhaps reprogramming impact of efferocytosis. Finally, it is important to note that future studies should not be limited to efferocytosis. Lung injury can trigger inflammatory modes of cell death, including ferroptosis and pyroptosis. Engulfment of these cell corpses shares machinery with AC clearance but engenders pro-inflammatory immune effects in engulfing phagocytes. 174 Collectively, efferocytosis remains a rich area of study with potential to facilitate a better understanding of repair and mis-repair processes. Deeper knowledge of the complex context in which cell death and clearance occur after tissue injury will be critical to developing effective, targeted therapeutics that facilitate repair.
